# Looking backward to move forward: a meta-analysis of stem cell therapy in amyotrophic lateral sclerosis

**DOI:** 10.1038/s41536-021-00131-5

**Published:** 2021-04-01

**Authors:** Cynthia Morata-Tarifa, Garikoitz Azkona, Jonathan Glass, Letizia Mazzini, Rosario Sanchez-Pernaute

**Affiliations:** 1 Andalusian Network for the Design and Translation of Advanced Therapies, Andalusian Health Ministry, Sevilla, Spain; 2 Department of Basic Psychological Processes and Their Development–School of Psychology, University of the Basque Country (UPV/EHU), San Sebastian, Spain; 3 Emory ALS Center, Neurology and Pathology Department, Emory University School of Medicine, Atlanta, GA USA; 4 ALS Centre, Department of Neurology, University Hospital “Maggiore della Carità”, Novara, Italy

**Keywords:** Stem-cell research, Regeneration and repair in the nervous system, Amyotrophic lateral sclerosis

## Abstract

Transplantation of several types of stem cells (SC) for the treatment of amyotrophic lateral sclerosis (ALS) has been evaluated in numerous Phase I/II clinical trials with inconclusive results. Here, we conducted a meta-analysis to systematically assess the outcome of SC therapy trials which report the evolution of each patient before and after cell administration. In this way, we aimed to determine the effect of the SC intervention despite individual heterogeneity in disease progression. We identified 670 references by electronic search and 90 full-text studies were evaluated according to the eligibility criteria. Eleven studies were included comprising 220 cell-treated patients who received mesenchymal (M) SC (*n* = 152), neural (N) SC (*n* = 57), or mononuclear cells (MNC: CD34, CD117, and CD133 positive cells) (*n* = 11). Our analyses indicate that whereas intrathecal injection of mesenchymal stromal cells appears to have a transient positive effect on clinical progression, as measured by the ALS functional rating score, there was a worsening of respiratory function measured by forced vital capacity after all interventions. Based on current evidence, we conclude that optimal cell product and route of administration need to be determined in properly controlled preclinical models before further advancing into ALS patients. In addition, in-depth understanding of disease mechanisms in subsets of patients will help tailoring SC therapy to specific targets and increase the likelihood of improving outcomes.

## Introduction

Amyotrophic lateral sclerosis (ALS) is a neurodegenerative disease with a largely unknown pathogenesis that primarily targets motor neurons. Recent studies report a prevalence between 4.1 and 8.4 per 100, 000 persons, with the male-to-female ratio being between 1 and 2. The mean age at diagnosis is 54–69 years and patients manifest weakness in the limbs (spinal onset) or difficulty in speaking or swallowing (bulbar onset) that leads to death, usually through respiratory failure, 24–50 months after the diagnosis^1^. About 10% of ALS cases are familial (FALS), caused by dominantly inherited autosomal mutations in *SOD1*, *TARDBP*, *FUS*, and *C9orf72*, among other genes^2^. Mutations in these genes, which also occur in sporadic forms of the disease, alter proteostasis, protein trafficking and quality control, perturb aspects of RNA stability, function and metabolism, nuclear pore transport, and cytoskeletal dynamics^3^. Neuropathological findings include degeneration and loss of motoneurons with astrocytic gliosis in the motor cortex, the brainstem, and the spinal cord, and, in most cases, ubiquitinated cytoplasmic TDP-43 inclusions. Besides, other pathogenic processes, such as glutamate toxicity, endoplasmic reticulum and oxidative stress, loss of trophic factors, inflammation, or mitochondrial dysfunction have been involved in disease onset and progression, based on experimental and clinical data^4^.

Despite much research, there has been little success in the quest for disease modifying or neuroprotective strategies, with only two FDA-approved pharmacological agents, riluzole and edaravone. Around 40% of SOD1 ALS patients have hyperexcitability and this correlates with a more severe outcome. Indeed, riluzole (2-amino-6-[trifluoromethoxy] benzothiazole, RP54274), the standard of care in ALS, is an antagonist of glutamatergic neurotransmission that has been demonstrated to slightly delay the onset of respiratory dysfunction and extend the median survival 2–3 months^5^. Edaravone (MCI-186, 3-methyl-1-phenyl-2-pyrazolin-5-one) is a strong antioxidant that has been recently shown to slow down the decline of ALS functional rating score (FRS) compared with placebo^6^. In addition, clinical trials are underway with antisense oligonucleotides targeting mutations in *SOD1* (NCT02623699)^7^ and *C9orf72* (NCT03626012)^8^ genes.

Stem cell (SC) therapy has been proposed as a promising treatment option for ALS based on the potential effects on various pathogenic mechanisms through trophic and/or immunomodulatory support and, perhaps, by providing host neural cell replacement^9^. Indeed, in the past two decades, transplantation of various SC products has been evaluated in numerous Phase I and II clinical trials designed to assess feasibility and safety, but also looking for indications of clinical benefit, reflected by changes in the progression rate of the ALS functional ratings score (ALSFRS) and the respiratory function (forced or slow vital capacity, F/SVC). Regarding efficacy, results are mostly inconclusive due to the limited sample size of the trials and the heterogeneity of individual progression. In addition, very few trials are controlled, as inclusion of adequate sham controls for invasive procedures is problematic and often unethical. Therefore, we decided to conduct a meta-analysis of SC trials considering all clinical trials that report the evolution of each patient before and after cell administration. In this way, we aimed to determine the efficacy of the SC administration despite individual heterogeneity.

## Results

### Selection of studies

**Fig. 1:**
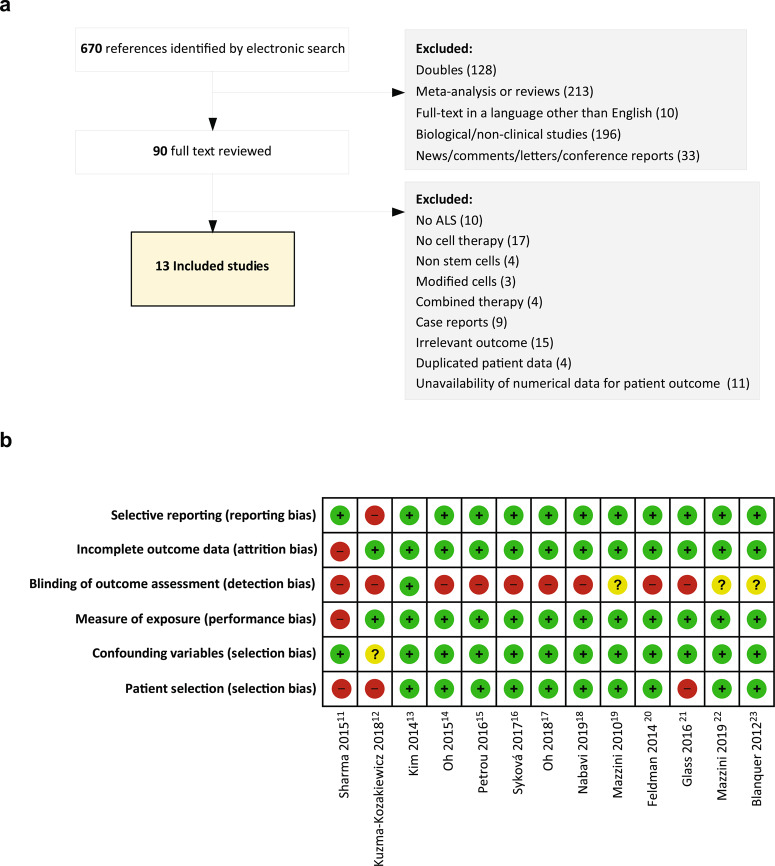
Meta-analysis design. **a** Selection of studies. **b** Risk of bias summary: authors’ judgment about each risk of bias item for the included studies. Red: high; yellow: unclear; green: low, according to RoBANS criteria^10^.

We systematically searched for references related to clinical trials using cell therapy in patients with ALS (Supplementary Table 1). We identified 670 references by electronic search and 90 full-text studies were evaluated according to the eligibility criteria. Eligibility criteria included reporting numerical data for ALSFRS and/or FVC at least for three time points: *t*_pre_, at ≥3 ± 0.5 months before the intervention, *t*_0_, at the time of the intervention (0 ≤ 0.5 months), and *t*_post_, at ≥3 months following the intervention, in order to calculate the scores for the lead-in and follow-up periods. Finally, 13 studies accomplished all eligibility criteria (Fig. 1a).

Following risk of bias assessment using the tool for non-randomized studies (RoBANS)^10^, another two studies^11,12^ were excluded due to a high risk of bias; only one study^13^ showed low risk for the six domains of RoBANS (Fig. 1b). Thus, 11 publications^13–23^ were included in the meta-analysis of which one was controlled^17^ and another one used historical controls^21^ in the analysis. The studies comprised 220 cell-treated patients who received mesenchymal (M) SCs (*n* = 152), neural (N) SCs (*n* = 57), or mononuclear cells (MNCs), which include CD34-, CD117-, and CD133-positive cells (*n* = 11). Cell dose and administration frequency varied among studies. The administration routes were intrathecal (six studies), intrathecal + intramuscular (one study), intravascular (one study), or intraspinal (five studies) (Table 1). Inclusion and exclusion criteria for the individual clinical trials are summarized in Supplementary Table 2. Mean baseline scores differed across individual studies, for ALSFRS-R, from 24.8 to 39.85 and for FVC, from >50% to 106%; (Supplementary Table 3).

**Table 1:** Clinical trials included in the meta-analysis.

Study	Patients (*n*)	Cell type	Flow cytometry	Dose: cells × 10^6^ × times	Administration route
Kim et al.^13^	Non-responders: 18	BM-MSC	CD29+, CD44+, CD49+, CD73+, CD105+ CD34−, CD45−	1/kg × 2 (1 month apart) in CSF	Intrathecal (L2–L3)
	Responders: 19				
Oh et al.^14^	7	BM-MSC	CD29+, CD44+, CD49+, CD73+, CD105+ CD34−, CD45−	1/kg × 2 (1 month apart) in CSF	Intrathecal (L2–L4)
Petrou et al.^15^	12	BM-MSC (NTF)	–	1 × 24 1/kg (single)	Intramuscular (biceps and triceps) Intrathecal
	14			Low dose: 1 + 1 × 24 Mid dose: 1.5 + 1.5 × 24 High dose: 2 + 2 × 24	Intrathecal + Intramuscular
Syková et al.^16^	26	BM-MSC	CD73+, CD90+, CD105+ CD34−, CD45−	15 ± 4.5 (single)	Intrathecal (lumbar)
Oh et al.^17^	Treated: 32	BM-MSC	CD29+, CD44+, CD73+, CD105+ CD34−, CD45−	1/kg × 2 (1 month apart) in CSF	Intrathecal (L2–L4)
	Controls: 27				
Nabavi et al.^18^	8	BM-MSC	CD44+, CD73+, CD90+, CD105+ CD34−, CD45−	2/kg in NaCl + alb 2%	Intrathecal
	6			2/kg in NaCl + alb 2%	Intravenous
Mazzini et al.^19^	10	BM-MSC	CD29+, CD44+, CD90+, CD105+, CD106+, CD166+ CD34−, CD45−	74.64 (11.4–120) in CSF	Intraspinal (T4–T6)
Feldman 2014^20^	15	Fetal NSC	NSI-566RSC	0.5 × 5 (unilateral/bilateral)	Intraspinal (C3–C5; L2–L4)
Glass et al.^21^	9	Fetal NSC	NSI-566RSC	0.5–1.5 × 5 (unilateral) 1 × 5 (bilateral)	Intraspinal (C3–C5; L2–L4)
	15			2 × 5 (bilateral) 4–8 × 10 (bilateral) 16 × 10 (bilateral) × 2	Intraspinal (C3–C5; L2–L4)
Mazzini et al.^22^	18	Fetal NSC	–	0.75 × 3 (unilateral/bilateral)	Intraspinal (C3–C5; T8–T11)
Blanquer et al.^23^	11	BM-MNC	CD34+, CD117+, CD133+	–	Intraspinal (T3–T4)

*BM* bone marrow, *MSC* mesenchymal stromal cells, *NTF* Neurotrophic factor, *CSF* cerebrospinal fluid, *alb* albumin.

### Effect of SC therapy on clinical score

**Fig. 2:**
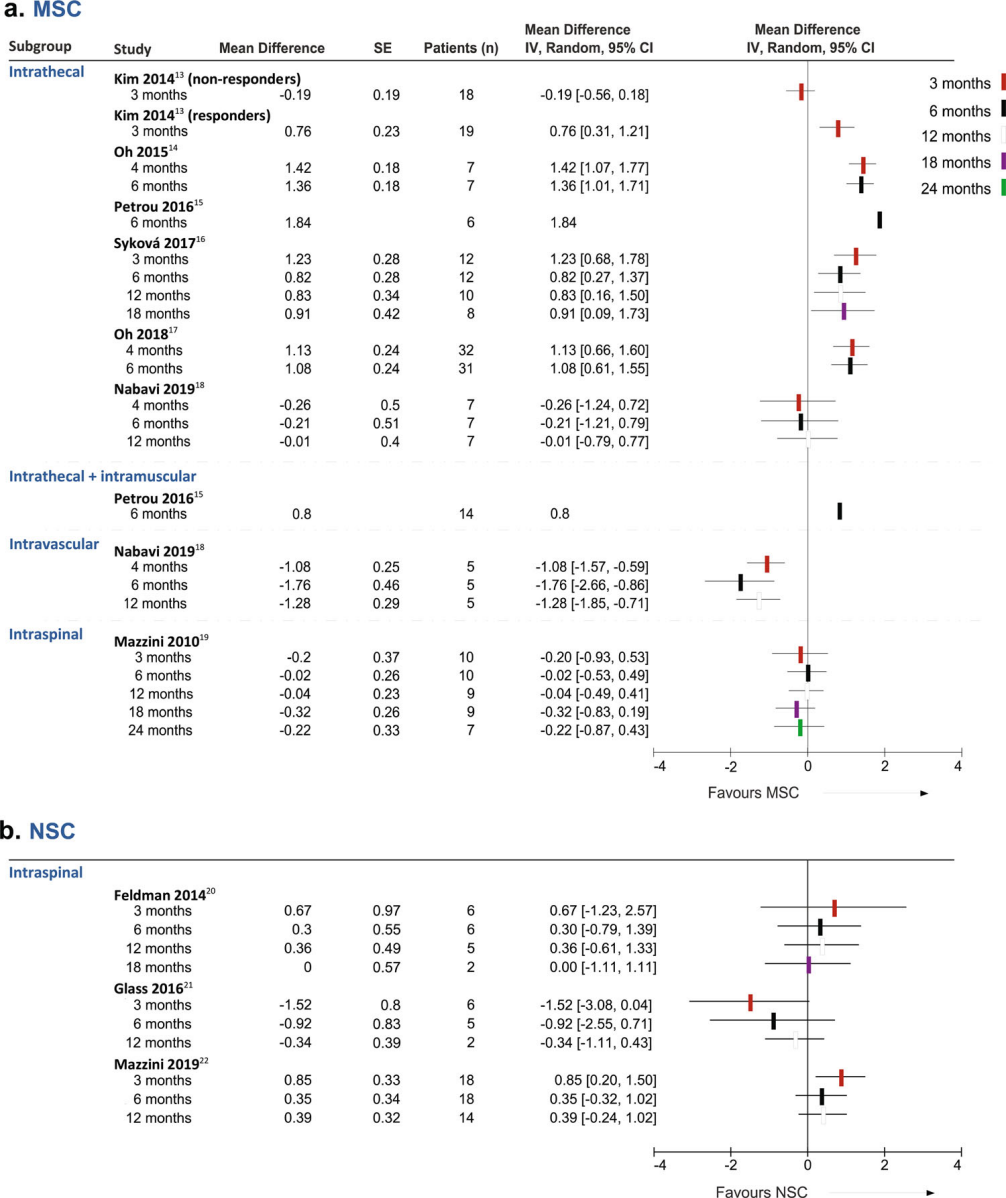
Effect of SC therapy on clinical score. Forest-plots of the changes in ALSFRS-R, in points per month, between the lead-in period and the follow-up [(score at *t*_*x*_ post − score at *t*_0_)/*x*] minus [(score at *t*_0_ − score at *t*_*X*_ pre)/*x*], evaluated at different follow-up time points, in patients infused with **a** MSCs or **b** NSCs. Squares and black lines represent the mean differences and 95% CI of individual studies and the different colors stand for the follow-up times, as indicated in the legend.

We first analyzed the evolution of patients included in each study, before and after cell administration comparing the score change for ALSFRS-R, in points per month (Table 2). Overall, MSC administration did not have a significant effect on ALSFRS-R decline rate, but with highly significant subgroup differences at all times examined. ALSFRS-R decline was significantly slower when the MSCs were injected intrathecally^14–18^ but not by intraspinal^19^ injection. The intravascular route^18^ was associated with a faster ALSFRS-R deterioration at all time points. The effects from forest-plot analysis of individual studies using MSCs are shown in Fig. 2a.

**Table 2:** Differences in ALSFRS-R change, in points per month, between the lead-in and follow-up periods.

	3–4 Months	6 Months	12 Months	18 Months	24 Months
*MSC*					
Intrathecal					
Patients (*n*)	95	57	17	8	NA
Subtotal (95% CI)	0.72 (0.12, 1.32)	0.92 (0.44, 1.40)	0.44 (−0.39, 1.26)	0.91 (0.09, 1.73)	NA
Test for overall effect;	*Z* = 2.36 *P* = 0.02	*Z* = 3.74 P = 0.0002	*Z* = 1.04 *P* = 0.30	*Z* = 2.17 *P* = 0.03	NA
Heterogeneity	*I*^2^ = 90%	*I*^2^ = 69%	*I*^2^ = 60%	NA	
Intrathecal + intramuscular					
Patients (*n*)	NA	14	NA	NA	NA
Subtotal (95% CI)	NA	0.80	NA	NA	NA
Test for overall effect;	NA	NA	NA	NA	NA
Heterogeneity					
Intravascular					
Patients (*n*)	5	5	5	NA	NA
Subtotal (95% CI)	−1.08 (−1.57, −0.59)	−1.76 (−2.66, −0.86)	−1.28 (−1.85, −0.71)	NA	NA
Test for overall effect;	*Z* = 4.32 *P* < 0.0001	*Z* = 3.84 *P* = 0.0001	*Z* = 4.41 *P* < 0.00001	NA	NA
Heterogeneity	NA	NA	NA		
Intraspinal					
Patients (*n*)	10	10	9	9	7
Subtotal (95% CI)	−0.2 (−0.93, 0.53)	−0.02 (−0.53, 0.49)	−0.04 (−0.50, 0.42)	−0.32 (−0.82, 0.18)	−0.22 (−0.87, 0.43)
Test for overall effect;	*Z* = 0.54 *P* = 0.59	*Z* = 0.08 *P* = 0.94	*Z* = 0.17 *P* = 0.86	*Z* = 1.25 *P* = 0.21	*Z* = 0.67 *P* = 0.51
Heterogeneity	NA	NA	NA	NA	NA
Total					
Patients (*n*)	110	72	31	17	NA
Total (95% CI)	0.37 (−0.29, 1.03)	0.28 (−0.48, 1.04)	−0.14 (−0.99, 0.71)	0.25 (−0.90, 1.45)	NA
Test for overall effect	*Z* = 1.11 *P* = 0.27	*Z* = 0.74 *P* = 0.46	*Z* = 0.32 *P* = 0.75	*Z* = 0.41 *P* = 0.68	NA
Test for subgroup differences	*P* < 0.0001	*P* < 0.00001	*P* = 0.0004	*P* = 0.01	NA
*NSC*					
Intraspinal					
Patients (n)	30	29	21	2	NA
Total (95% CI)	0.07 (−1.43, 1.57)	0.19 (−0.35, 0.74)	0.15 (−0.33, 0.62)	0.00 (−1.11, 1.11)	NA
Test for overall effect;	*Z* = 0.09 *P* = 0.93	*Z* = 0.70 *P* = 0.49	*Z* = 0.61 *P* = 0.54	*Z* = 0.00 *P* = 1.00	NA
Heterogeneity	*I*^2^ = 74%	*I*^2^ = 2%	*I*^2^ = 13%	NA	
*MNC*					
Intraspinal					
Patients (*n*)	11	11	9	NA	NA
Total (95% CI)	0.47 (−0.39, 1.33)	0.56 (−0.30, 1.42)	0.41 (−0.41, 1.23)	NA	NA
Test for overall effect;	*Z* = 1.07 *P* = 0.29	*Z* = 1.28 *P* = 0.20	*Z* = 0.98 *P* = 0.33	NA	NA
Heterogeneity	NA	NA	NA		

*NA* not applicable.

The analysis of three NSC^20–22^ and one MNC^23^ studies showed that the intraspinal administration did not have a significant effect on ALSFRS-R decline rate (Table 2). Regarding NSC injection, not all the patients included in two of the trials^20,21^ had sufficient data to calculate the scores for the lead-in period and were excluded. It is also important to note that the number of included patients treated with MNCs was very small. The effects from forest-plot analysis of individual studies using NSCs are shown in Fig. 2b. These were all administered by direct injection into the spinal cord.

### Effect of SC therapy on pulmonary function

**Fig. 3:**
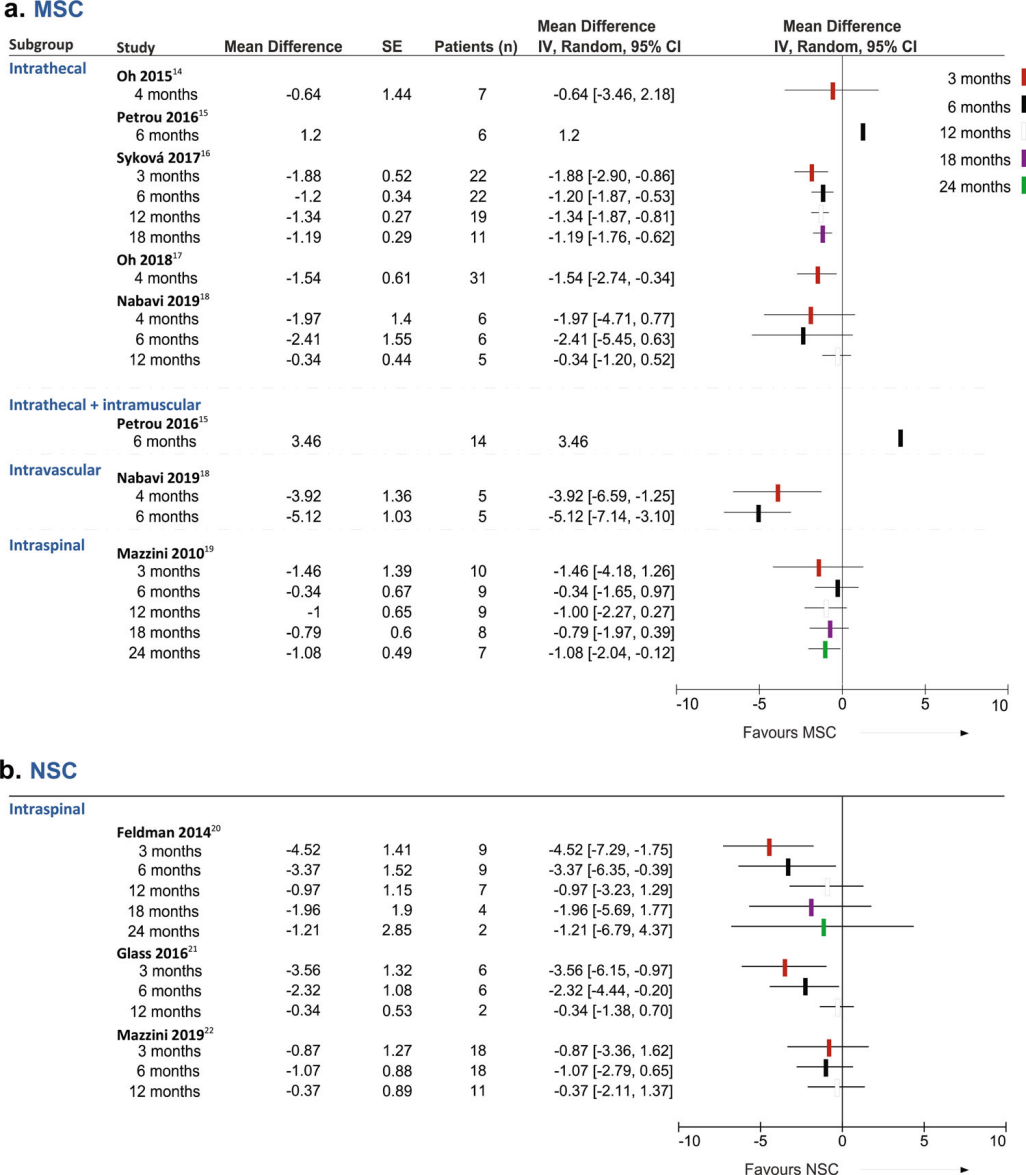
Effect of SC therapy on pulmonary function. Forest-plots of the changes in FVC, in points per month, between the lead-in period and the follow-up values [(score at *t*_*x*_ post − score at *t*_0_)/*x*] minus [(score at *t*_0_ − score at *t*_*x*_ pre)/*x*], evaluated at different time points in patients infused with **a** MSCs or **b** NSCs. Squares and black lines represent the mean differences and 95% CI of individual studies and the different colors stand for the follow-up time as indicated in the legend.

FVC decline was significantly faster in the post-treatment period compared with the lead-in period for patients treated with MSCs (Table 3). Subgroup analysis showed that the administration of MSCs by intrathecal route^14–18^ at 3, 6, and 18 months, by intraspinal^19^ route at 24 months, or by intravascular route^18^ at all times was associated with a faster FVC decline. Intriguingly, the intrathecal + intramuscular (biceps and triceps) administration^15^ of MSCs in 14 patients was associated with an improvement in the FVC post-treatment, but we did not have access to the raw data for this study. The effects on FVC from forest-plot analysis of individual studies using MSCs are shown in Fig. 3a. FVC decline was significantly steeper at 3 and 6 months post-treatment compared with the lead-in period in patients infused with NSCs^20–22^ as well (Fig. 3b). Patients infused with NSCs showed an improvement at later times, but this was probably influenced by the loss of most severe patients (Table 3). A similar pattern was observed in patients infused with MNCs^23^ (Table 3).

**Table 3:** Differences in FVC change per month between the lead-in and follow-up periods.

	3–4 Months	6 Months	12 Months	18 Months	24 Months
*MSC*					
Intrathecal					
Patients (*n*)	66	28	24	11	NA
Subtotal (95% CI)	−1.68 (−2.40, −0.96)	−1.26 (−1.91, −0.60)	−0.90 (−1.87, 0.07)	−1.19 (−1.76, −0.62)	NA
Test for overall effect; Heterogeneity	*Z* = 4.57 *P* <0.00001	*Z* = 3.78 *P* = 0.0002	*Z* = 1.81 *P* = 0.07	*Z* = 4.10 *P* <0.0001	NA
	*I*^2^ = 0%	*I*^2^ = 0%	*I*^2^ = 73%	NA	
Intrathecal + intramuscular					
Patients (*n*)	NA	14	NA	NA	NA
Subtotal (95% CI)	NA	3.46	NA	NA	NA
Test for overall effect; Heterogeneity	NA	NA	NA	NA	NA
Intravascular					
Patients (*n*)	5	5	NA	NA	NA
Subtotal (95% CI)	−3.92 (−6.59, −1.25)	−5.12 (−7.14, −3.10)	NA	NA	NA
Test for overall effect; Heterogeneity	*Z* = 2.88 *P* = 0.004 NA	*Z* = 4.97 *P* < 0.00001 NA	NA	NA	NA
Intraspinal					
Patients (*n*)	10	9	9	8	7
Subtotal (95% CI)	−1.46 (−4.18, 1.26)	−0.34 (−1.65, 0.97)	−1.00 (−2.27, 0.27)	−0.79 (−1.97, 0.39)	−1.08 (−2.05, −0.11)
Test for overall effect; Heterogeneity	*Z* = 1.05 *P* = 0.29 NA	*Z* = 0.51 *P* = 0.61 NA	*Z* = 1.54 *P* = 0.12 NA	*Z* = 1.32 *P* = 0.19 NA	*Z* = 2.18 *P* = 0.03 NA
Total					
Patients (*n*)	81	42	33	19	NA
Total (95% CI)	−1.81 (−2.49, −1.14)	−2.07 (−3.81, −0.32)	−0.95 (−1.61, −0.29)	−1.11 (−1.63, −0.60)	NA
Test for overall effect	*Z* = 5.26 *P* < 0.00001	*Z* = 2.32 *P* = 0.02	*Z* = 2.83 *P* = 0.005	*Z* = 4.27 *P* < 0.0001	NA
Test for subgroup differences	*P* = 0.27	*P* = 0.0004	*P* = 0.90	*P* = 0.55	NA
*NSC*					
Intraspinal					
Patients (*n*)	33	33	20	4	2
Total (95% CI)	−2.92 (−5.09, −0.76)	−1.87 (−3.09, −0.65)	−0.43 (−1.27, 0.40)	−1.96 (−5.69, 1.77)	−1.21 (−6.79, 4.37)
Test for overall effect; Heterogeneity	*Z* = 2.65 *P* = 0.008 *I*^2^ = 51%	*Z* = 3.00 *P* = 0.003 *I*^2^ = 0%	*Z* = 1.02 *P* = 0.31 *I*^2^ = 0%	*Z* = 1.03 *P* = 0.30 NA	*Z* = 0.43 *P* = 0.67 NA
*MNC*					
Intraspinal					
Patients (*n*)	11	11	9	NA	NA
Total (95% CI)	−0.91 (−3.20, 1.38)	0.18 (−1.94, 2.30)	0.07 (−2.13, 2.27)	NA	NA
Test for overall effect; Heterogeneity	*Z* = 0.78 *P* = 0.44 NA	*Z* = 0.17 *P* = 0.87 NA	*Z* = 0.06 *P* = 0.95 NA	NA	NA

*NA* not applicable.

To rule out publication bias, we performed funnel plots. Egger’s test showed no asymmetry indicative of publication bias for the MSC studies: *P* = 0.5 for ALSFRS (*k* = 8) and *P* = 0.78 for FVC (*k* = 6) at 3 months. For the NSC meta-analysis, inspection of the funnel plot distribution did not support a correlation between the effect size and the sample (Egger’s test neither showed a significant asymmetry, *P* = 0.55 for ALSFRS and *P* = 0.27 for FVC, but with only three studies regression lacks sensitivity).

### Subgroup analyses

Given that the meta-analysis revealed strong effect of subgroups, but because of the heterogeneity of trial designs we could not compare each condition side by side, we decided to perform additional two-way analyses of variance to examine the effects of the cell product and the route of administration on the progression of ALSFRS-R and FVC, using the slopes calculated from all available scores for the lead-in period and the follow-up of individual studies (Supplementary Fig. 1).

**Fig. 4:**
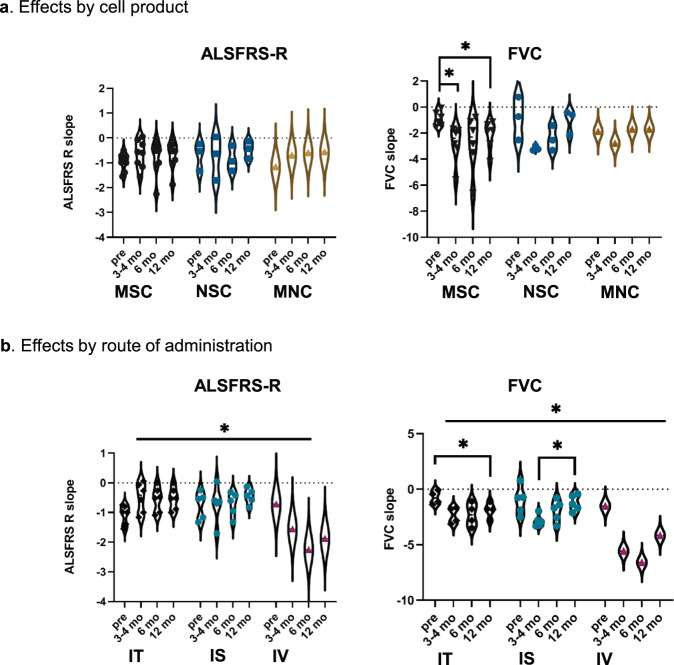
Effects of cell type and delivery on outcomes. Violin plots showing the effect of **a** the cell product and **b** the administration route, on the differences between ALSFRS-R and FVC slopes calculated for the lead-in period (pre) and the follow-up time points, as indicated. Points represent individual studies. Discontinued lines represent the mean value. Two-way ANOVA with post-hoc Tukey’s test, *P ≤ 0.05. IS intraspinal, IT intrathecal, IV intravascular, mo month.

Regarding the cell product—and taking into account that there were highly significant differences between the studies within the three groups, i.e. MSC, NSC, and MNC (*P* = 0.0034)—we found no significant effect on the ALSFRS-R slopes. There was neither a significant effect of the cell product on the FVC slopes, but in this case we could detect a significant effect of time (*P* = 0.028) with significant differences in the MSC group between the slopes in the lead-in period and the follow-up at 3–4 months (*P* = 0.043) and at 12 months (*P* = 0.03) (Fig. 4a).

We next examined the effect of the route of administration on ALSFRS-R, and detected a significant effect of the route (*P* = 0.0508) with significant interaction of time and route (*F*_6,27_ = 3.163, *P* = 0.0176) but no significant differences within groups in the post-hoc analysis. On the other hand, the effect of the route on the FVC was significant (*F*_2,7_ = 8.07, *P* = 0.015) showing a faster progression for all routes, with a highly significant effect of time and a significant interaction of time and route. In the post-hoc analysis, the differences between the slopes for the lead-in period and the follow-up were significant at 12 months for intrathecal, *P* = 0.025 and showed a trend for intraspinal, *P* = 0.09. The post-hoc analysis also showed a difference between 3 and 12 months for the intraspinal route (*P* = 0.05) but in the opposite direction (improvement) (Fig. 4b), consistent with the forest-plot results.

### Adverse events

Finally, although all included studies were considered to be safe, we examined the adverse events (AEs) related to the intervention, as a relevant aspect in risk-management for cell therapies. AEs are presented in Supplementary Table 4. Most common AEs were general disorders and events related with the administration site (45.05 % [91/202]), nervous system disorders (40.1% [81/202]), and musculoskeletal and connective tissue disorders (19.3% [39/202]). We further analyzed the frequency of AEs by route of administration (Supplementary Fig. 2). Two trials with intraspinal NSC injections reported serious AEs related to the treatment. In the first, a Phase I study^20^, three patients out of six reported wound dehiscence (*n* = 1), incisional (*n* = 2) and neck (*n* = 1) pain, and headache (*n* = 1) that were resolved. In the second, a Phase II trial^21^, one of the patients experienced neurologic deterioration due to acute spinal cord swelling, and another one suffered neuropathic pain. Importantly, none of these were completely resolved.

## Discussion

SC therapy is viewed as a promising option for ALS because these cells potentially target several of the putative pathogenic mechanisms involved in the onset and progression of the disease, although explicit target engagement for each cell product needs further validation^9^. Here, we designed a meta-analysis to examine individual progression as a measure of efficacy, using the ALSFRS-R change, which is considered to be linear for the majority of the illness^24^ Overall, and with the limitations that are discussed below, we found that, based on current evidence, cell therapy has a transient positive effect on the progression of the disease, as measured by the ALSFRS-R. In the available controlled studies that we reviewed (76 patients receiving MSCs or MNCs), there was also a trend for a better progression in patients receiving cell therapy. In those studies, the administration of MSCs was intrathecal^17^ or intrathecal + intramuscular^25^, and intracortical for MNCs^26^.

In our meta-analysis, the effect of the SC therapy on ALSFRS-R was not huge when considering the monthly score but, if sustained, the cumulative effect could represent a meaningful clinical change at 6 months and later. For context, note that the difference at 6 months for the FDA-approved antioxidant edaravone over placebo was 2.41 points in a selected cohort^27^, or ~0.41 points per month. Additional considerations need to be made because (1) some studies reported a very short follow-up, (2) patients included in some of these trials were in an advanced stage (and this precluded us to analyze the effect on survival) and, (3) long-term changes, although encouraging, need to be interpreted with caution and are more difficult to associate with the intervention due to loss of the most severe patients during the study. Notwithstanding, the clinical data supports a positive effect of the intervention on the progression of the symptoms, if transient.

We also examined the effect on FVC, as a value less likely to be biased. However, and unlike ALSFRS-R, the progression of FVC has been shown to follow distinct trajectories^28^, so the comparison of pre- and post-treatment FVC slopes maybe less informative with respect to the effect of the intervention. Also, the slope calculation was less accurate as there were fewer values reported and fewer studies reporting values. Nevertheless, we cannot ignore that, overall, there was a worsening of FVC values after the intervention that was pronounced early on. The most plausible interpretation is that the intervention disrupts a delicate respiratory functional balance, being, in that regard, not different from other surgical interventions that appear to accelerate the progression of ALSFRS^29^. However, this is an unlikely explanation for the intrathecal administration, which is a minor procedure and did not cause significant worsening in a controlled study^27^ raising questions about potential MSC off-target effects. Indeed, in the controlled study^27^, the authors reported that the FVC progression in points per month was not significantly different between controls (−0.8 ± 3.18, *n* = 25) and MSC-treated patients (−1.54 ± 3.38, *n* = 31) with respect to their lead-in periods, i.e. a difference of −0.74 (−2.52, 1.03) points per month in favor of controls, but note that MSC-treated patients showed a significant worsening in the follow-up period with respect to the lead-in (*P* = 0.023) while controls did not (*P* = 0.29). On the other hand, for intraparenchymal interventions, the effect of the SC treatment on ALSFRS-R could be larger if compared with a sham procedure, emphasizing the inadequacy of non-interventional controls and the need to investigate these issues in properly controlled preclinical models.

The source and class of the cells to be transplanted represent an arguable point for the implementation of cell therapy in central nervous system (CNS) disorders. Most clinical trials—and all the intrathecal ones—included in this meta-analysis were performed with autologous Bone marrow mesenchymal stromal cells (BM-MSCs). These trials presented some differences regarding cell preparation, cell dose, and administration regime and, overall, those using intrathecal repeated administration in cerebrospinal fluid vehicle appear the most promising. The rational to use MSCs in ALS is based on their capacity to produce and release neurotrophins^30^ and secrete molecules that can suppress the activation and function of both the innate and adaptive immune systems^31^. In ALS patients, increased numbers of CD4+ and CD8+ T cells and dendritic cells have been detected near dying motoneurons in the spinal cord and in brain parenchyma^32^. Preclinical studies have reported that human MSC transplantation attenuates neuroinflammation, improves motor performance, and extends survival in a mouse model of ALS^33^. In addition, it is possible that mitochondrial transfer from MSCs can improve bioenergetics of recipient cells^34^. However, a precise characterization of the biodistribution of MSCs and definition of the mechanism(s) of action is lacking. Since BM-MSCs were obtained from the same patient in these trials, there was no need to immunosuppress the patients. However, patient-derived SCs may express genetic and epigenetic disease-related footprints, making them unsuitable for therapeutic purposes^35^. Nevertheless BM-MSCs from ALS patients have been reported to maintain the cytokine secretory profile and to be more efficient in decreasing TNF alpha—but also to respond differently to induction^36^ (somewhat questioning NurOwn^®^ strategy)^15,25^.

Initially driven to achieve neuronal cell replacement, fetal NSCs were embraced as a cell source that is renewable while being inherently non-tumorigenic and with low immunogenicity^37^. Nonetheless, NSCs appear more likely to differentiate into glial cells (astrocytes and oligodendrocytes) and to provide trophic support to dying motoneurons^35^. A toxic effect of astrocytes has been well established in ALS spinal cord, therefore, providing healthy glia into the area may have a positive impact on disease outcome. The fetal NSCs used in these studies had different origins in the spinal cord (Neuralstem Inc.)^20,21^ or forebrain^22^ which may define their restorative capacity, as regional differences in glial cells are becoming increasingly recognized^38^. Immunosuppression is also a factor that needs to be considered when using allogeneic cells, even if fetal NSCs appear to induce little immunogenicity^39^ and survival has been documented after withdrawal of immunosuppression^40^. In that postmortem analysis, nests of transplanted spinal NSCs were identified but did not show significant differentiation into glia and only subsets were labeled for SOX2 or NeuN (a marker of mature neurons)^40^. Indeed, protective mechanisms may be mediated by the NSCs or by other cell types as Blanquer et al. reported the presence of nests of MNC transplanted cells surrounding motoneurons which showed less ubiquitin inclusions and were more numerous at targeted levels (if only ~25% from control)^23^. With currently available datasets, these three SC types can only be compared in intraspinal administration, but the observed effects are too small to draw any conclusion.

For any therapy, but in particular for those targeting the CNS, because of the barriers and enclosures that protect it, delivery is critical. For ALS, local delivery targeting the upper motoneurons (intracortical), lower motoneurons (intraspinal), or neuromuscular junction (intramuscular) requires invasive procedures and multiple injections, each representing a risk of AEs, which, in particular, into the CNS parenchyma may have serious consequences, and therefore requires a careful risk–benefit analysis. Equally important, with local approaches the cell distribution is rather restricted to the injected segment or area (for example, with 20 intraspinal injections only about 15% (8 cm) of the spinal cord area was covered)^41^. This has to be taken into account when calculating the cell dose because increasing the cell number without increasing their spread could be detrimental.

On the other end, systemic (intravenous) delivery of MSCs while convenient from a logistic point of view has been found to be mostly ineffective^42^.

Thus, intrathecal delivery by injection into the subarachnoid space presents the most favorable profile, achieving a broader distribution and, possibly, penetration in the parenchyma through paravascular spaces, while using relatively minor invasive procedures. This route makes it feasible to repeat the administration and to perform a sham procedure allowing to evaluate separately the effect of the procedure and that of the SC product (although in this case all trials that used the intrathecal route were done with BM-MSCs so it is not possible to completely separate the effect of both variables on the outcome). A more recently proposed infusion into the subpial space^41^ appears promising in preclinical models, by achieving a broad distribution of infused cells, but requires surgery making more problematic to repeat the administration. Given that the effect on the clinical progression appears to be transient, an invasive procedure would not be desirable.

A compelling lesson learned from previous research is that confirmatory trials of the efficacy of new SC lines for the treatment of ALS should be initiated only after an evidence-based treatment protocol is constructed. There are distinct challenges and opportunities in performing early-phase SC therapy trials in ALS, which require special approaches and unique trial designs. The current stage of the clinical research in this field requires to identify a primary outcome measure which can show an improvement that is clinically meaningful to the patient so that the risk of the procedure is reasonably balanced by the potential clinical benefits and the value of the resulting scientific knowledge. Investigators should choose to stratify for factors most relevant to the outcome measure of the trial. Patient selection is a very important variable, but to date, there is little agreement about inclusion criteria. A more heterogeneous study population may mask efficacy of the study intervention on specific subpopulations of patients bearing genetic forms of the disease or restricted phenotypes. According to the updated guidelines for ALS clinical trial design^43^, responder analyses should be included, having the potential to demonstrate beneficial effects of a proposed treatment on a subset of patients with a shared unique phenotype. Moreover, in designing and implementing ALS SC clinical trials, investigators should incorporate predictive biomarkers, prognostic biomarkers, and, especially biomarkers of SC activity.

Overall, based on current evidence, we can conclude that SC therapy may have a positive, if transient, effect on ALS progression, but not on the pulmonary function, and that both the cell product and the delivery route need to be carefully reconsidered and optimized in adequately controlled preclinical models before moving forward in ALS patients. Furthermore, for a SC therapy to be successful, a third factor, the recipient, needs also to be integrated in the equation. Illustrating this point, the results of NurOwn^®^ Phase III trial (NCT03280056) in which patients received three intrathecal injections of autologous pre-conditioned BM-MSCs or placebo were released last month. The trial did not meet the endpoint but analysis of a subgroup of early patients showed the anticipated difference between treated and placebo groups. The company has announced initiation of expanded access program (compassionate use) for some patients who completed the clinical trial and meet specific eligibility criteria (https://ir.brainstorm-cell.com/2020-11-17-BrainStorm-Announces-Topline-Results-from-NurOwn-R-Phase-3-ALS-Study). Thus, a better understanding of disease mechanisms in individual patients would greatly enhance the possibility to implement the most suitable SC strategy to modify the prognosis of ALS patients.

## Methods

### Search strategy and selection criteria

This systematic review and meta-analysis is reported according to the PRISMA statement. We comprehensively searched for full-text published studies with no publication date restriction. Studies reporting data related to the administration of human SCs for the treatment of patients with ALS were identified using MEDLINE. The last search was carried out on June 30, 2020. The search strategy is detailed in Supplementary Table 1. Only full-text clinical studies published in English, containing data for ALSFRS and/or FVC, were included. We excluded meta-analysis and reviews, and case reports.

### Data extraction and quality assessment

Study selection was performed by C.M-T, G.A., and R.S-P, examining independently the full text of potentially relevant studies and applying eligibility criteria to select the included studies, by consensus. Eligibility criteria included reporting numerical data for relevant outcomes, ALSFRS and/or FVC, for at least three time points: *t*_pre_, at ≥3 ± 0.5 months before the intervention, *t*_0_, at the time of the intervention (0 ≤ 0.5 months), and *t*_post_, at ≥3 months following the intervention, in order to calculate the scores for the lead-in and follow-up periods. Information obtained from these studies enclosed inclusion criteria, a description of the patients, characteristics of the administered treatment, and the outcomes. Communication with the author of the included studies was established in cases of doubt or to request additional data. C.M-T. extracted the outcome data from the included studies and G.A. and R.S-P. revised the extracted data. Disagreements were solved by discussion and consensus between the authors. To evaluate the methodological quality of the included studies, we employed the risk of bias assessment tool for non-randomized studies (RoBANS)^10^, since most of the included studies were non-randomized. Note that not all patients who participated in one of the 11 included studies had sufficient data to be used in the meta-analysis and slope calculations. Actual numbers are provided in Tables 2 and 3.

### Outcomes

The following outcomes were assessed: (1) ALSFRS-R and (2) FVC, before and after treatment, and (3) incidence of AEs.

### Statistical analysis

We carried out the statistical analysis using Review Manager 5.3 (RevMan 5.3). Each data point is a study (not a patient). Subgroup analyses included cell type and administration route. Mean differences, SD, and 95% confidence interval (CI) were calculated for changes in the monthly rate of decline of ALSFRS-R and FVC between the lead-in period and the follow-up periods for each patient. The scores (mean and SD) collected as ALSFRS were normalized and recalculated for a 48-point scale, in order to analyze together the point-per-month and slopes for all studies. We used inverse-variance to estimate the intervention effect. Random-effects model was used to consider the differences between individual study effects to estimate the treatment effect. Between-study variance was calculated using the DerSimonian–Laird method^44^. The heterogeneity between studies was assessed using *I*^2^ test. Funnel plots and Egger’s test were employed to assess publication bias^45^ using dmetar package in *R* v4.0.3.

Non-linear regression (least squares fit) was used to calculate the pre- and post-treatment slopes for ALSFRS-R and FVC from all available data points of individual studies (mean and SD). Two-way analysis of variance (ANOVA) tests grouped by cell product or route of delivery were used to compare the effects of the intervention on the slopes at different times, followed by post-hoc Tukey’s multiple comparisons test. These analyses were performed using GraphPad Prism v8.4.3. Statistical significance was considered when *P* ≤ 0.05.

### Reporting summary

Further information on research design is available in the Nature Research Reporting Summary linked to this article.

## Supplementary information

Supplementary Information

Reporting Summary Checklist

## Data Availability

Aggregated data used in the study are available from the corresponding author upon reasonable request.
