# Supplementary material for: Looking backward to move forward: a meta-analysis of stem cell therapy in amyotrophic lateral sclerosis
Source: NPJ Regen Med. 2021 Apr 1;6:20. doi: 10.1038/s41536-021-00131-5 (PMC8016966; doi:10.1038/s41536-021-00131-5)
Supplement: Supplementary file 1 — Supplementary Information [file 41536_2021_131_MOESM1_ESM.pdf]

Supplementary Online Content

Looking backwards to move forward: A meta-analysis of stem cell therapy in Amyotrophic Lateral Sclerosis

Supplementary Table 1. Search strategy

Supplementary Table 2. Inclusion/exclusion criteria employed in the included studies

Supplementary Table 3. General characteristics of the included studies

Supplementary Table 4. Adverse events from included studies

Supplementary Figure 1. Pre- and post-treatment slopes for AFLSFRS-R and FVC

Supplementary Figure 2. Percentage of adverse events by type and administration route

Supplementary Table 1. Search strategy

Medline		
Database(s): Medline (No publication date restriction).<br>Search Strategy:		
**#**	**Searches**	**Results**
1	"ALS"[All Fields] AND "Stem Cell"[All Fields] AND (Clinical Study[ptyp])	36
2	"ALS"[All Fields] AND "Cell Transplantation"[All Fields] AND (Clinical Study[ptyp])	39
3	"Amyotrophic Lateral Sclerosis"[All Fields] AND "Mesenchymal Stromal Cell"[All Fields] AND (Clinical Study[ptyp])	16
4	"ALS"[All Fields] AND "Mesenchymal Stromal Cell"[All Fields] AND (Clinical Study[ptyp])	13
5	"Amyotrophic Lateral Sclerosis"[All Fields] AND "Mononuclear Cell"[All Fields] AND (Clinical Study[ptyp])	11
6	"ALS"[All Fields] AND "Mononuclear Cell"[All Fields] AND (Clinical Study[ptyp])	12
7	"Amyotrophic Lateral Sclerosis"[All Fields] AND "Neural Stem Cell"[All Fields] AND (Clinical Study[ptyp])	11
8	"ALS"[All Fields] AND "Neural Stem Cell"[All Fields] AND (Clinical Study[ptyp])	9
9	"Amyotrophic Lateral Sclerosis"[All Fields] AND "Cell Transplantation"[All Fields]	523

Supplementary Table 2. Inclusion/exclusion criteria

Study	Inclusion criteria	Exclusion criteria
Kim 2014[13]	• Clinical diagnosis of probable or definite ALS based on the El Escorial World Federation of Neurology criteria<br>• ALSFRS-R score ≥ 20 points<br>• Age between 25 and 65 years<br>• Ability to visit the clinic alone or with a caregiver's support<br>• Informed consent	• High protein levels or lymphocytosis in the CSF<br>• Positive titers of anti-GM1 antibodies<br>• Severe conduction blocks or slow conduction velocities<br>• Medical or inflammatory disorders markedly interfering with the ability to interpret the results of the study<br>• Active infections.
Oh 2015[14]	• Patients between 25–75 years of age<br>• Diagnosed with clinically probable or definite ALS according to the revised El Escorial criteria<br>• ALSFRS-R score between 31 and 46<br>• Riluzole treatment at the stable dose (50 mg, twice daily) for at least 3 months prior screening<br>• Disease duration no longer than 5 years prior to the first diagnosis	• Participation in other clinical trials<br>• FVC < 40% of the predicted value<br>• Presence of any concomitant disease that might interfere with the outcome (neurological disease other than ALS, psychiatric disorders, cancer, systemic disease, cardiovascular disease, hepatic or renal disorder, or any other disease)<br>• Tracheostomal ventilation or NIV for more than 12 hours per day<br>• Hemorrhagic tendency at the time of screening<br>• Administration of any drug that could affect the BM
Petrou 2016[15]	• El Escorial criteria for definite or probable ALS<br>• Either men or non pregnant women between 20-75 years of age<br>• Patient is mentally intact and psychologically stable<br>• For early stage ALS- Patients will be with ALSFRS-R scale of at least 30 and disease duration of less than 2 years, or for progressive stage ALS- Patients will be with an ALS-FRS-R scale of 15-30 and disease duration of less than 2 years<br>• For early stage ALS- Patient has sufficiently bulky muscles. Or, for progressive stage ALS- Patient with at least 60% FVC<br>• Participant understands the nature of the procedure and provides written informed consent prior to any study procedure	• Positive test for HBV, HCV, HIV and Mycoplasma<br>• High protein in the CSF<br>• Lymphocytosis in the CSF<br>• Positive for anti-GM1 antibodies<br>• Patient has significant conduction blocks or slow nerve conduction velocities (a reduction of more than 30%) confirmed by nerve conduction velocity - EMG studies<br>• The patient is a respiratory dependent<br>• Renal failure, impaired hepatic function<br>• Patients suffering from significant cardiac disease, malignant diseases or any other disease that may risk the patient or interfere with the ability to interpret the results<br>• Active infections<br>• Participation in another clinical trial within 1 month prior to start of this study<br>• Subject unwilling or unable to comply with the requirements of the protocol<br>• Patient has not been treated previously with any cellular therapy
Syková 2017[16]	• Diagnosis of definite ALS according to: El Escorial Revised criteria, data available from detailed neurological observations, ALSFRS, Norris scale, FVC, brain and spinal cord MRI for at least 6 months prior to the study commencement to exclude pretreatment pathology such as tumor or spine stenosis<br>• Riluzole-naive or on a stable dose for at least 2 months<br>• Male or female aged between 18 and 65 years<br>• Life expectancy of more than 2 years	• FVC < 70%<br>• Paralysis < 15 points on the Norris bulbar scale in case of primary bulbar, < 15 points on the Norris spinal scale<br>• Pregnancy<br>• Breastfeeding<br>• Coagulopathy<br>• Skin infection at the site of BM aspiration or administration of the cell product<br>• Gastrostomy<br>• Medical condition that could compromise the safety of the patient (e.g., recent myocardial infarction, congestive heart failure, renal failure, liver failure, cancer, systemic infection, recurrent thromboembolic disease)<br>• Alcohol or drug abuse<br>• Women of childbearing potential not using effective contraception
Oh 2018[17]	• Patients between 25–75 years of age<br>• Diagnosed with clinically probable or definite ALS according to the revised El Escorial criteria<br>• ALSFRS-R score between 31 and 46<br>• Stable riluzole treatment (50 mg, twice daily) for at least 3 months before screening<br>• Disease duration no longer than 5 years after the onset of the first symptom	• Participation in other clinical trials within the past 12 months<br>• FVC < 40% of the predicted value<br>• Presence of any comorbidity that might interfere with the outcome<br>• Tracheostomy or NIV<br>• Hemorrhagic tendency<br>• Administration of any drug that could affect the BM
Nabavi 2019[18]	• Definitive diagnosis of sporadic ALS due to EL Escorial criteria,<br>• 24-60 years<br>• More than 6 months of evolution of disease<br>• ALSFRS score ≥ 24<br>• FVC ≥ 40%<br>• Riluzole, at a dose of 100 mg, twice per day	• Any concomitant neurological, psychiatric or systemic diseases<br>• Use of any corticosteroids, immunoglobulin, or immunosuppressant treatments during 6 months before enrollment
Mazzini 2010[19]	• Patients between 20 and 65 years old<br>• Definite or probable sporadic ALS according to the El Escorial Revised Criteria<br>• Spinal onset<br>• Duration of the disease less than 3 years<br>• Evidence of progression disease in the last 6 months<br>• Mild to moderate disability documented by satisfactory bulbar and spinal function (minimal score of 3 on ALSFRS for swallowing, and 2 for cutting food and handling utensils, and walking)	• Patients over 65<br>• Patients with familial ALS<br>• Evidence of any concurrent illness (diabetes, cardiovascular disorders, arterial hyper-tension, kidney and liver disorders, dysthyroidism, autoimmune disorders, neoplasms or any other diseases reducing life expectancy)<br>• Receiving any medications which might affect BM

	• FVC ≥ 50% of that predicted<br>• Normal polysomnography<br>• Good acceptance and under-standing of the informed consent	
Feldman 2014[20]	• Ability to understand the requirements of the study, provide written informed consent, understand and provide written authorization for the use and disclosure of PHI [per HIPAA Privacy Ruling] and comply with the study procedures<br>• Sporadic or familial ALS diagnosed as laboratory-supported probable, probable or definite according to the World Federation of Neurology El Escorial Criteria, based on examination by the site PI<br>• Age 18 years or older<br>• Females must have a negative serum pregnancy test and practice an acceptable method of contraception or be of non-childbearing potential (post-menopausal for at least 2 years or surgically sterile [hysterectomy, oophorectomy or surgical sterilization])<br>• Geographic accessibility to the study center and the ability to travel to the clinic for study visits<br>• Presence of a willing and able caregiver<br>• Medically able to undergo lumbar or cervical laminectomy as determined by the Investigator, surgeon and anesthesiologist<br>• Medically able to tolerate immunosuppression regimen consisting of basiliximab, tacrolimus, mycophenolate mofetil, and methylprednisolone as determined by the site Investigator<br>• Agrees to the visit schedule as outlined in the informed consent<br>• Not taking riluzole (Rilutek®) or on a stable dose for ≥ 30 days<br>• All required vaccinations current: tetanus/diptheria (TDAP), herpes zoster/shingles (Vostavax®: within last 10 years and must be prior to surgery), pneumonia (Pneumovax®), seasonal/H1N1 flu vaccines (as appropriate for season) for Groups B-E	• Etiology of paraplegia or weakness is due to causes other than ALS such as spinal ischemia, traumatic spinal injury, traumatic brain injury, multiple sclerosis, cerebral stroke, cerebral palsy, or infection<br>• VC < 60% predicted normal by standard nomogram at the time of screening and VC < 50% predicted normal measured supine for age at the time of surgery<br>• Current or peak PRA due to alloantibodies > 20% receiving their first allograft<br>• Any known immunodeficiency syndrome<br>• Receipt of any investigational drug, device or biologic within 30 days of surgery<br>• Any concomitant medical disease or condition limiting the safety to participate:<br>o Coagulopathy<br>o Active uncontrolled infection<br>o Hypotension requiring vasopressor therapy<br>o Previous spinal surgery at the site of planned transplantation except for ACDF<br>o Skin breakdown over the site of surgery<br>o Malignancy (except for non-melanoma skin cancer)<br>o Primary or secondary immune deficiency<br>o Spinal stenosis<br>• Creatinine > 1.5, liver function tests (SGOT/SGPT, Bilirubin, Alk Phos) > 2x the upper limit of normal, hematocrit/hemoglobin < 30/10, total WBC < 4000, uncontrolled hypertension (defined as systolic > 180 or diastolic > 100) or uncontrolled diabetes (defined as hemoglobin A1C > 8), evidence of GI bleeding by hemoccult test, positive tuberculosis (TB test: PPD/Mantoux), hepatitis B or C, or HIV<br>• Presence of any of the following conditions:<br>o Current drug abuse or alcoholism<br>o Unstable medical conditions<br>o Unstable psychiatric illness including psychosis and untreated major depression within 90 days of screening<br>o Positive blood test for hepatitis B or C<br>o Any condition that the site PI feels may interfere with participation in the study<br>o Any condition that the surgeon feels may pose complications for the surgery<br>o Known hypersensitivity to basiliximab, tacrolimus, mycophenolate mofetil, or methylprednisolone<br>• Inability to provide informed consent as determined by screening protocol<br>Inadequate family or caregiver support as determined by the site PI.
Glass 2016[21]	• Extremity weakness and/or spasticity due to ALS<br>• Ability to understand the requirements of the study, provide written informed consent, understand and provide written authorization for the use and disclosure PHI [per HIPAA Privacy Ruling] and comply with the study procedures<br>• Sporadic or familial ALS, meeting the definition of laboratory-supported probable, probable or definite ALS according to the World Federation of Neurology El Escorial Criteria<br>• At the time of consent subjects should be within 24 months of symptom onset. At the time of surgery, subjects should be within 36 months of symptom onset<br>• Age 18 years or older<br>• Females must have a negative serum pregnancy test and practice an acceptable method of contraception or be of non-childbearing potential (post-menopausal for at least 2 years or surgically sterile [hysterectomy, oophorectomy or surgical sterilization])<br>• Geographic accessibility to the study center and the ability to travel to the clinic for study visits<br>• Presence of a willing and able caregiver<br>• Medically able to undergo cervical and lumbar (Group E only) laminectomy or laminoplasty<br>• Subjects with positive toxoplasmosis IgG agrees to take Bactrim DS 1 tab PO 3 times weekly during the 6-month treatment period<br>• Medically able to tolerate the immunosuppression regimen consisting of basiliximab, tacrolimus, mycophenolate mofetil, prednisone and methylprednisolone<br>• Agrees to the visit schedule as outlined in the informed consent.<br>• Not taking riluzole (Rilutek®) or on a stable dose for ≥ 30 days prior to surgery<br>• VC ≥ 60% of predicted normal for age, height and gender measured in the seated position and ≥ 50% in supine position at screening and at ≤ 7 days prior to surgery	

Mazzini 2019[22]	• Patients aged 20-75 years<br>• Definitive diagnosis of ALS with spinal onset (according to El Escorial-Revised criteria)<br>• Lower motor neuron degeneration in at least three regions of the cord (cervical, thoracic, and lumbosacral)<br>• Progressive weakness attributed to ALS over the last 6 months<br>• FVC ≥ 60% of their predicted value<br>• Not using invasive respiratory support	• Psychiatric diseases or neurological diseases other than ALS<br>• Mental deterioration or cognitive disturbances<br>• Unable to understand the informed consent form and study aims<br>• Evidence of concurrent illness<br>• Receiving corticosteroids, immunoglobulin, or immunosuppressive treatment
Blanquer 2012[23]	• Patients between 20 and 65 years old<br>• Definite ALS according to the El Escorial criteria<br>• Spinal onset<br>• Duration of disease between 6 and 36 months<br>• FVC ≥ 50% of that predicted<br>• < 90% fall in oxygen saturation (T90) occurring in ≤ 2% of sleep time.	• Evidence of concomitant neurological, psychiatric, or systemic disease<br>• Treatment with corticosteroids, immunoglobulins, or immunosuppressors in the last 12 months<br>• Included in another clinical trial<br>• Required enteral or parenteral nutrition<br>• Pregnancy<br>• Unable to provide informed consent

ACDF, anterior cervical dissection fusion; **ALS**, amyotrophic lateral sclerosis; **ALSFRS**, ALS functional rating scale; **ALSFRS-R**, ALS functional rating scale-revised; **BM**, bone marrow; **CSF**, cerebrospinal fluid; **EMG**, Electromyography; **FVC**, forced vital capacity; **HIPAA**, health insurance portability and accountability act; **HBV**, hepatitis B; **HCV**, hepatitis C; **HIV**, human immunodeficiency virus; **IT**, intrathecal; **MRI**, magnetic resonance imaging; **NIV**, noninvasive ventilation; **PHI**, protected health information; **PRA**, panel reactive antibody; **VC**, vital capacity.

Supplementary Table 3. General characteristics of the included studies

Study	Country	Type of study	Control group	Patients (n) Gender (M/F)	Age (yr) (Range)	Site of onset	Disease duration at entry (mo) (Range)	ALSFRS-R Score (Range)	FVC (%) (Range)	Follow up duration (mo)
Kim 2014[13]	Korea	Phase I	Responders	19 (12/7)	52.7 ± 11.9	Spinal (94.7%)<br>Bulbar (5.3%)	24.2 ± 13.4	36.4 ± 5.1	67.1 ± 16.4	6
			Nonresponders	18 (8/10)	48.8 ± 8.8	Spinal (88.8%)<br>Bulbar (11.1%)	24.1 ± 14.1	38.2 ± 3.9	72.4 ± 22.8	6
Oh 2015[14]	Korea	Phase I	No	7 (2/5)	45.71 ± 9.63 (29-62)	Spinal (71.4%)<br>Bulbar (28.6%)	22.86 ± 8.35 (13-35)	38.29 ± 1.16 (32-40)	77.71 ± 7.4 (58-86)	12
Petrou 2016[15]	Israel	Phase I/II	No	12 (8/4)	48.1 ± 12.9 (30-65)	-	-	*IT:* 24.8 ± 2.5 (22-28)<br>*IM:* 34.8 ± 4.3 (30-42)	> 50	6
		Phase II	No	14 (8/6)	50.8 ± 11.4 (23-64)	-	-	39.85 ± 2.9 (34-42)	> 50	
Syková 2017[16]	Czech Republic	Phase I/II	No	26 (14/12)	51.2 ± 1.7 (33-64)	Spinal (50%)<br>Bulbospinal (50%)	38.1 ± 4.2 (11-99)	29.5 ± 1 (15-37)*	88.8 ± 2.3 (70-118)	18
Oh 2018[17]	Korea	Phase II	Treated	32 (18/14)	53.7 ± 7.7	Spinal (71.9%)<br>Bulbar (28.1%)	11.7 ± 8.8	35.5 ± 4.2	73.1 ± 21.1	6
			Controls	27 (11/16)	52.5 ± 9.4	Spinal (77.7%)<br>Bulbar (22.3%)	24 ± 11.5	34.7 ± 5.5	71.3 ± 16.1	
Nabavi 2019[18]	Iran	Phase I	No	*IT:* 8 (7/1)<br>*IV:* 6 (2/4)	*IT:* 43.88 ± 11.16 (24-57)<br>*IV:* 49.17 ± 1 0.8 (34-60)	-	*IT:* 19.88 ± 13.66<br>*IV:* 13.17 ± 5.55	*IT:* 31.14 ± 4.12 (24-38)*<br>*IV:* 29.8 ± 3.48 (26-34)*	*IT:* 76.66 ± 16.75 (45.2-95)<br>*IV:* 83 ± 7.94 (72-95)	12
Mazzini 2010[19]	Italy	Phase I	No	10 (7/3)	41.8 ± 11.82 (20-61)	Spinal	11.9 ± 9.79 (3-30)	33 ± 3.77 (26-39) *	93.6 ± 19.36 (51-117)	24
Feldman 2014[20]	USA	Phase I	No	15 (13/2)	50.86 ± 9.6 (35-66)	-	45.67 ± 41.47	-	-	20
Glass 2016[21]	USA	Phase I	No	9 (7/2)	49.76 ± 9.2 (35-65)	Spinal (88.8%)<br>Bulbar (11.2%)	42.1 ± 38.38 (15-141)	33.11 ± 3.8 (29-40)	93.11 ± 13.94 (74-125)	24
		Phase II	No	15 (12/3)	49.36 ± 9.82 (29-66)	Spinal (93.3%)<br>Bulbar (6.7%)	15.49 ± 5.32 (5-23)	37.33 ± 5.37 (28-47)	89.27 ± 12.3 (70-110)	
Mazzini 2019[22]	Italy	Phase I	No	18 (15/3)	48.39 ± 11.49 (25-67)	-	-	34.33 ± 6.77	84.94 ± 13.98	60
Blanquer 2012[23]	Spain	Phase I	No	11 (5/6)	46.76 ± 7.1 (31-61)	Spinal (100%)	24.5 ± 8.34 (14-40)	30.83 ± 4.02 (24-38) *	106.25 ± 11.48 (79-121)	12

ALSFRS, ALS functional rating scale; **ALSFRS-R**, ALS functional rating scale-revised; **F**, female; **IT**, intrathecal; **IM**, intramuscular; **IV**, intravascular; **M**, male; **Mo**, months; **Yr**, years. *indicates studies reporting ALSFRS

Supplementary Table 4. Adverse events from included studies

Studies	Patients (n)	General disorders and administration site conditions	Musculoskeletal and connective tissue dissorders	Gastrointestinal disorders	Nervous system disorders	Respiratory disorders	Others
		**Adverse events (number of patients, %)**					
		**Intrathecal**					
		**MSC**					
**Kim 2014**[13]	37	Fever 11 (29.73)	Low back pain 4 (10.81)<br>General mialgia 9 (24.32)	0 (0)	Headache 4 (10.81)	0 (0)	0 (0)
**Oh 2015**[14]	8	Pyrexia 3 (37.5)<br>Pain 2 (25)	0 (0)	0 (0)	Headache 2 (25)	0 (0)	0 (0)
**Sykova 2017**[16]	26	0 (0)	0 (0)	0 (0)	Headache 7 (26.92)	0 (0)	Hyperhydrosis 1 (3.84)<br>Leukocytosis 2 (7.69)
**Oh 2018**[17]	33	Pyrexia 1 (3.03)<br>Pain 1 (3.03)	0 (0)	0 (0)	Headache 2 (6.06)	0 (0)	0 (0)
**Nabavi 2019**[18]	7	0 (0)	0 (0)	Nausea and vomiting 2 (28.57)	Headache 2 (28.57)	0 (0)	0 (0)
		**Intrathecal and intramuscular**					
		**MSC-NTF**					
**Petrou 2016**[15]	26	Fever 11 (42.31)<br>Bruising 1 (3.85)	Back/leg pain 8 (30.77)<br>Neck stiffness 2 (7.69)<br>General weakness 1 (3.85)	Vomiting 3 (11.54)	Headache 13 (50)<br>Spasticity 1 (3.85)	0 (0)	0 (0)
		**Intravascular**					
		**MSC**					
**Nabavi 2019**[18]	5	Hypotension 1 (20)	0 (0)	0 (0)	0 (0)	0 (0)	0 (0)
		**Intraspinal**					
		**MSC**					
**Mazzini 2010**[19]	10	Pain 7 (70)	0 (0)	0 (0)	Tingling 6 (60)<br>Sensory light-touch impairment in one leg 4 (40)<br>Sensory light-touch impairment in sacral region 1 (10)	0 (0)	0 (0)
		**NSC**					
**Feldman 2014**[20]	6	Wound dehiscence 1 (16.67)<br>Incisional pain 6 (100)<br>Neck pain 3 (50)<br>Grinding in neck 1 (16.67)	Cervical kyphosis 1 (16.67)<br>Shoulder pain 1 (16.67)	Constipation 1 (16.67)<br>Nausea/vomiting 1 (16.67)	Muscle spam 3 (50)<br>Headache 3 (50)	Laryngeal edema 1 (16.67)<br>Hiccups 2 (33.33)	Urinary retention 1 (16.67)<br>Popliteal vein thrombosis 1 (16.67)
**Glass 2016**[21]	15	Incision pain 12 (80)<br>Nausea secondary to surgery 1 (6.67)<br>Fall 1 (6.67)<br>Other injury or procedural complications 3 (20)	Muscle pain/ache 3 (20)<br>Neck pain/stiffness 7 (46.67)<br>Other 3 (20)	Diarrhea 2 (13.33)<br>Nauseas/vomiting 3 (20)<br>Constipation 5 (33.33)<br>Duodenitis 1 (6.67)<br>Other 2 (13.33)	Muscle weakness 2 (13.33)<br>Headache 2 (13.33)<br>Hypoesthesia 6 (40)<br>Neuralgia 3 (20)<br>Paresthesia 4 (26.67)<br>Spinal cord edema 1 (6.67)<br>Myoclonus 1 (6.67)	Pleural effusion 1 (6.67)	Blood disorders 1 (6.67)<br>Infections:<br>UTI 2 (13.33)<br>Pneumonia 3 (20)<br>Other 3 (20)<br>Disorders of metabolism and nutrition:

					Tremor 2 (13.33)<br>Insomnia 2 (13.33)<br>Other 1 (6.67)<br>Hallucination 1 (6.67)		Acute diabetes mellitus 2 (13.33)<br>Hyperglycemia 3 (20)<br>Abnormal liver function tests 2 (13.33)<br>Other 2 (13.33)<br>Renal/urinary disorders<br>Urinary incontinence 1 (6.67)<br>Other 2 (13.33)<br>Skin disorders<br>Erythema 1 (6.67)<br>Other 2 (13.33)<br>Vascular disorders<br>DVT 2 (13.33)<br>Other 1 (6.67)<br>Crepitus 1 (6.67)<br>Fatigue 1 (6.67)<br>Ventricular tachycardia 1 (6.67)
**Mazzini 2019**[22]	18	Pain 8 (44.44)<br>Hematoma at the site of surgical scare 1 (5.55)	0 (0)	0 (0)	Tremor 1 (5.55)<br>Tingling sensation 2 (11.11)<br>Spasms in the lower limbs 1 (5.55)	Acute respiratory failure 1 (5.55)<br>Pneumonia 4 (22.22)	Iatrogenic diabetes 1 (5.55)<br>Deep vein thrombosis 1 (5.55)
		**MNC**					
**Blanquer 2012**[23]	11	Pain 5 (45.45)<br>Painful wound 7 (63.63)<br>Intracranial hypotension 3 (27.27)<br>Transplant site hyperesthesia 1 (9.09)	0 (0)	Constipation 10 (90.91)	Headache 2 (18.18)<br>Insomnia 1 (9.09)<br>Vertigo 1 (9.09)	Hypoesthesia 7 (63.63)<br>Paresthesia 4 (36.36)<br>Dysesthesia 2 (18.18)	0 (0)

Supplementary Figure 1a.

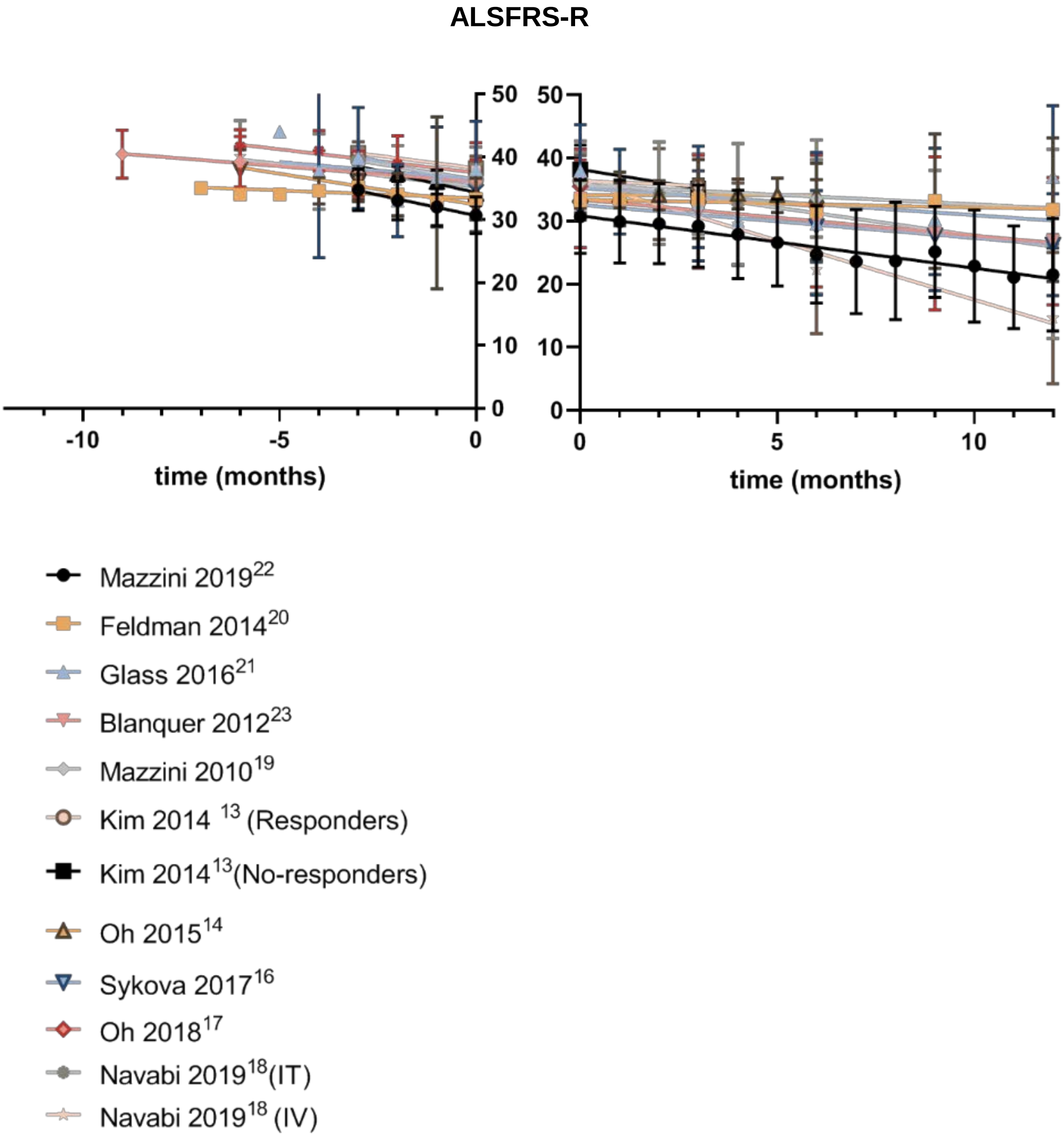

a. AFLSFRS-R pre- and post-treatment slopes. Mean and SD with regression lines are plotted for each study.

Supplementary Figure 1b.

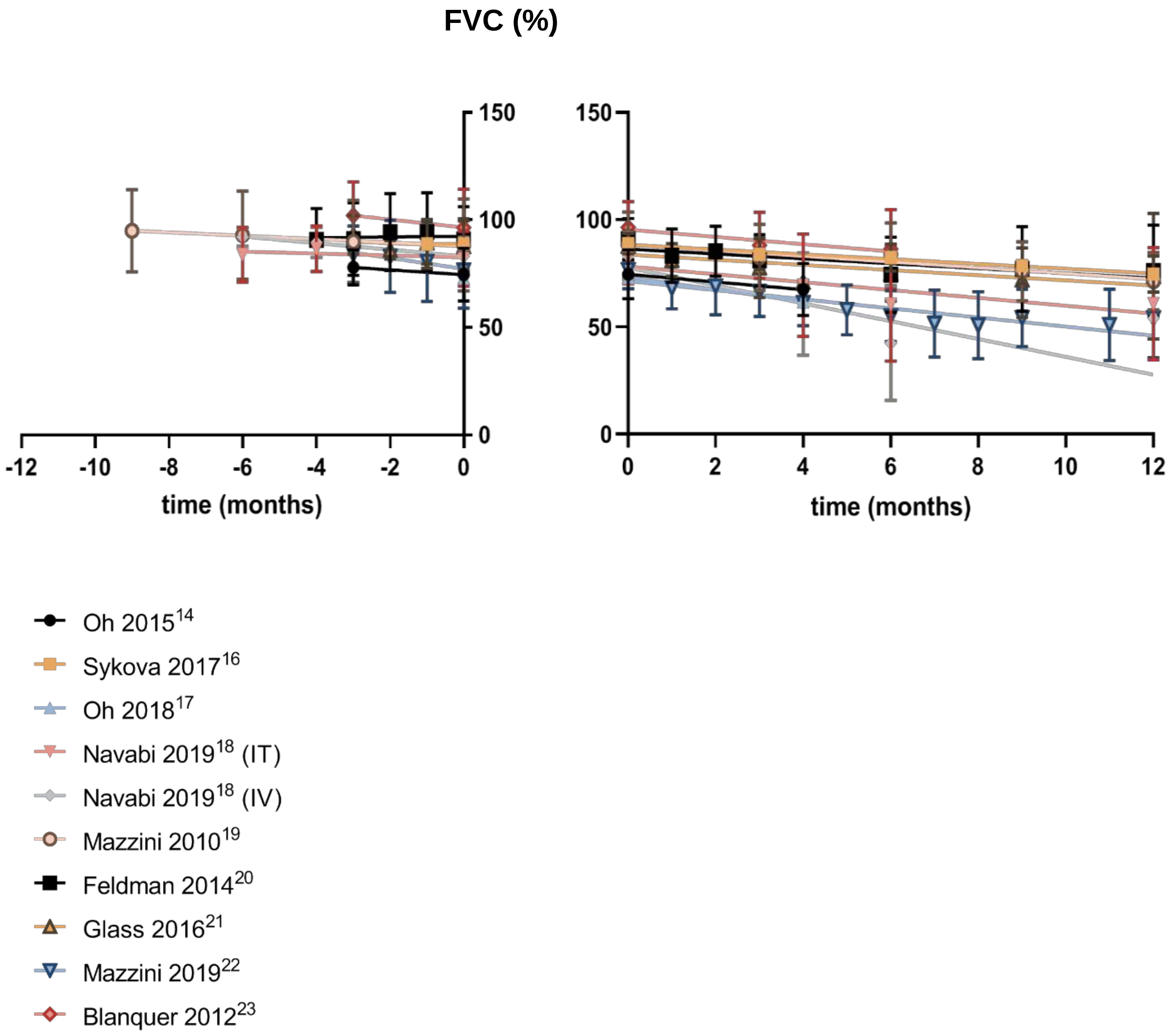

b. FVC pre- and post-treatment slopes. Mean and SD with regression lines for the studies included in the meta-analysis.

Supplementary Figure 2.

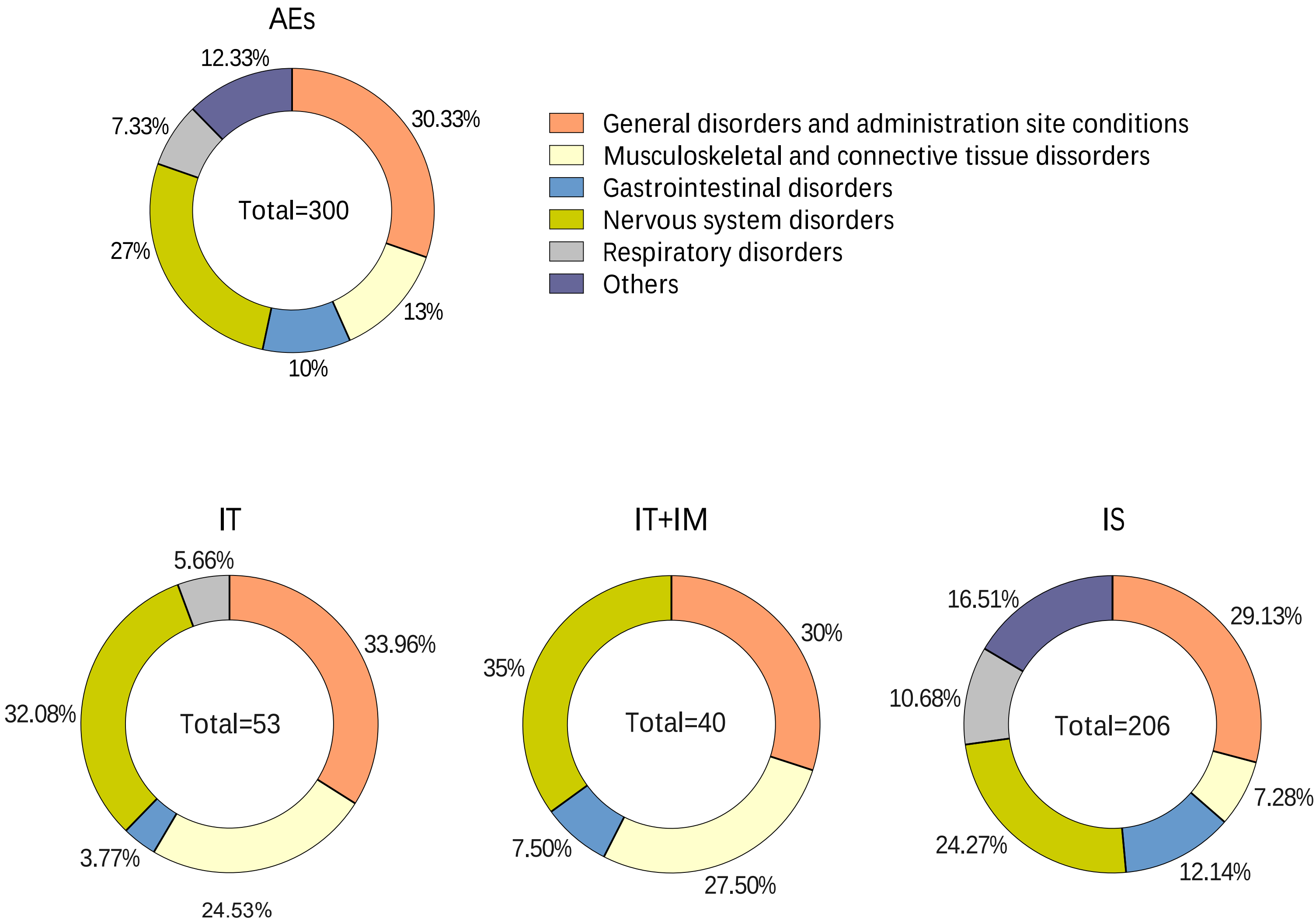

Percentage of adverse events by type of event and by administration route. IT: intrathecal, IM: intramuscular, IS: intraspinal.
